# Evaluation of serum and tissue levels of cold-inducible RNA-binding protein in non-segmental Vitiligo

**DOI:** 10.1007/s00403-023-02586-6

**Published:** 2023-03-15

**Authors:** Nayera Hassan Moftah, Huda Alnos, Laila Rashed, Mervat Hamdino

**Affiliations:** 1 Dermatology and Venereology Department, Faculty of Medicine for Girls, Al-Azhar University, Cairo, Egypt; 2 Biochemistry Department, Faculty of Medicine, Cairo University, Cairo, Egypt

**Keywords:** Non-segmental vitiligo, CIRP, Autoimmune diseases

## Abstract

Damage-associated molecular patterns (DAMPs) play a role in the pathogenesis of vitiligo. It has been established that the cold-inducible RNA-binding protein (CIRP), a member of the family of cold-shock proteins that respond to stress, is a DAMP molecule that promotes inflammation. The objective was to evaluate the serum and tissue CIRP expression in non-segmental vitiligo (NSV) patients. A sample of 40 participants, 20 NSV patients and 20 control groups of age- and sex-matched healthy individuals were included in this case–control study where the enzyme-linked immunosorbent assay was used in detecting the serum and tissue CIRP levels in participants. The serum and tissue CIRP levels significantly increased in NSV patients compared with the healthy controls, (165.35 ± 24.42, 226.29 ± 24.00 versus 59.81 ± 12.10, 105.86 ± 11.27 pg/ml, respectively) (*P* < 0.01). Serum and tissue CIRP are significantly correlated with each other (*r* = 0.641, *P* = 0.002). Except for a statistically significant positive correlation between CIRP tissue level and VASI (*r* = 0.539, *P* = 0.014), the CIRP Serum and tissue did not show any statistically significant correlations with different clinical parameters in patients. ROC curve shows that the cut-off point for serum and tissue CIRP level to differentiate between patients and controls was 86.5, 124.3 pg/ml, respectively, with 100.0% sensitivity, 100.0% specificity and 1.000 AUC for each of them. It is concluded that CIRP may have a crucial role in the pathogenesis of NSV and could be used as a marker for vitiligo and its extent with the need for further large-scale study.

## Introduction

Vitiligo is considered an acquired pigmentary disorder that impacts 0.5 to 2% of people worldwide across the sexes in addition to all races with an unpredictable course [1]. Vitiligo, despite having no impact on life expectancy, is linked to substantial quality-of-life (QoL) deficits in psychological health, ordinary lifestyle, and employment [2].

Vitiligo is a disorder that relies on multiple factors, and to destroy the melanocyte, several mechanisms have been proposed. That may be genetic, oxidative stress, melanocyte detachment and autoimmune theories. It appears that the adaptive and innate immune systems together are concerned [3]. Vitiligo may be associated with systemic autoimmune diseases like autoimmune thyroiditis, lupus erythematosus, alopecia areata and scleroderma [4].

Cold-inducible RNA-binding protein (CIRP), one of the cold shock proteins, is an evolutionarily conserved RNA chaperone that is broadly spread at low concentrations in diverse cells and tissues [5]. CIRP expression is significantly upregulated when exposed to cellular stress, such as hypothermia, hypoxia, or ultraviolet radiation, and after that transmigrates from the nucleus to the cytoplasm to perform its protecting roles in messenger RNAs stabilization and processing [6].

Previous research shows that CIRP may behave as DAMP when secreted extracellularly, activating inflammation and injury as it adhered to the TLR4-MD2 complex on professional antigen-presenting cells (APCs) and induced IL-6, HMGB1 and TNF-α release [7].

The role of DAMPs in vitiligo was previously demonstrated that under oxidative stress, melanocyte release DAMPs, which activate dendritic cells via pattern recognition receptors (PRRs) for presenting melanocyte-specific antigens to T cells results in the autoimmune destruction of melanocytes [8]. Several DAMPs were found in the perilesional skin of vitiligo subjects, including high mobility group box 1 (HMGB1) and heat shock protein70 (Hsp70) [9, 10].

However, the inclusions of CIRP in vitiligo have not been cleared. Therefore, this study aimed to assess the levels of CIRP in patients with NSV.

## Patients and methods

The committee of research ethics of the Faculty of Medicine for Girls, Al-Azhar University has approved the study protocol and informed consent form. All participants signed a written informed consent form.

### Participants

This case–control study was performed on patients visiting Al-Zahraa University Hospital’s Dermatology Outpatient Clinics, from October 2021 to July 2022. 20 adult patients with NSV of both genders were compared to 20 healthy age- and sex-matched controls (HCs), who were not first-degree relatives of the patients and attended for cosmetic purposes. The study excluded participants who had a history of malignancy, autoimmune disease, pregnancy, lactation, topical, phototherapy or systemic treatment of vitiligo one month before the study or any other conditions known to elevate CIRP levels.

### Clinical assessment

Every participant was subjected to a thorough history-taking, a complete dermatological and general examination that included disease activity evaluation according to Vitiligo Disease Activity (VIDA) score [11], and Vitiligo Area Scoring Index (VASI) for vitiligo extent assessment [12].

### Sample preparation

#### Blood samples

Three millilitres of venous blood were taken under sterile considerations, from each participant, after being left for two hours at room temperature, the collected samples were centrifuged for 20 min at 1000×*g* approximately. Before analysis, serum samples were kept at − 80 ℃.

#### Tissue samples

As for patients, a two-mm punch-skin biopsy was taken from non-exposed sites at the margin of active vitiliginous lesions of the patients (by considering the history of the disease progression or extension of old lesions and by using wood’s light to detect the actual margin) or from the margin of the latest lesion. As for healthy controls, a biopsy was taken from a matching area to the vitiliginous lesions of the patients. The supernate was obtained by centrifuging the tissue homogenates for 5 min at 5000×*g*. The samples were saved at 80 °C before testing.

### ELISA analysis of tissue and serum CIRP levels

Through an ELISA kit (MyBioSource, San Diego, California, USA, Catalog No: MBS761988), the CIRP levels were determined in serum and tissue homogenate in accordance with the manufacturer’s instructions. The wells were filled with the standards, test samples, and biotin-conjugated detection antibody before being rinsed with wash buffer. HRP-Streptavidin is then added, and the unbound conjugates are removed with a wash buffer. TMB substrates were applied to visualize the HRP enzymatic process, which was catalysed by HRP to produce a blue product that became yellow when an acidic stop solution was added. The quantity of the CIRP sample that was collected and the intensity of the yellow colour are correlated. After reading the O.D. absorbance at 450 nm in a microplate reader, the CIRP level could be calculated.

### Statistical analysis

Data were collected, revised, coded, and entered to the Statistical Package for Social Science (IBM SPSS) version 23 (SPSS Inc. Chicago, Il, USA. The quantitative data were presented as mean, standard deviations and ranges when parametric and median, inter-quartile range (IQR) when data were found non-parametric. In addition, qualitative variables were presented as number and percentages. The comparison between two groups with quantitative data and parametric distribution was done by *Independent t test* while the comparison of categorical data was done by Chi-square test. Correlation analysis was done by Pearson correlation. Receiver operation characteristic was done to detect sensitivity, specificity, positive and negative predictive values. *P* value < 0.05 is considered statistically significant.

## Results

This study enrolled 20 NSV patients and 20 healthy controls. 10 (50%) out of 20 NSV patients were males with an age range from 18 to 50 years old. Regarding the control group, 8 (40%) were males with an age range from 22 to 51 years old. Patients’ clinical data together with a comparison to healthy controls are illustrated in Table 1.

**Table 1:** clinical data of NSV patients and Healthy controls

Parameters	Groups		Significance	
	Patients	HCs	Test value	*P* value
Age				
Mean ± SD	41.55 ± 10.62	40.55 ± 9.56	*t* = 0.313	0.404
Range	18–50	22–51		
Sex				
Male	10 (50.0%)	12 (60.0%)	*X*^*2*^ = 0.404	0.525
Female	10 (50.0%)	8 (40.0%)		
Skin phenotype				
III	3 (15.0%)	4 (20.0%)	*X*^*2*^ = 0.173	0.677
IIII	17 (85.0%)	16 (80.0%)		
Family history				
Negative	16 (80.0%)			
Positive	4 (20.0%)			
Vitiligo duration (years)				
Median (IQR)	10.5 (4–20)			
Range	0.6–40			
VASI				
Median (IQR) Range	15.1 (1.5–35.5) 0.6–99.1			
VIDA, *n* (%)				
0	3 (15.0%)			
+ 1	4 (20.0%)			
+ 2	4 (20.0%)			
+ 3	6 (30.0%)			
+ 4	3 (15.0%)			

*HC* healthy control, *IQR* interquartile range, *VASI* Vitiligo Area Scoring Index, *VIDA* Vitiligo disease activity

A significant statistical elevation in CIRP serum and tissue levels in NSV patients was found in comparison with healthy controls with *p* value < 0.001 in each of them (Table 2).

**Table 2:** Comparison between patients group and control group regarding CIRP serum and tissue levels

	Patients (*n*: 20)	Healthy controls ( *n*: 20)	Test value•	*P* value
Serum CIRP (pg\ml)				
Mean ± SD	165.35 ± 24.42	59.81 ± 12.10	17.319	< 0.001*
Range	124.8–201.3	34.2–86.5		
Tissue CIRP (pg/ml)				
Mean ± SD	226.29 ± 24.00	105.86 ± 11.27	20.311	< 0.001*
Range	192.6–273.1	83.4–124.3		

*CIRP* cold-inducible RNA-binding protein

•Independent *t* test

^*^P value < 0.05: is considered statistically significant

**Fig. 1:**
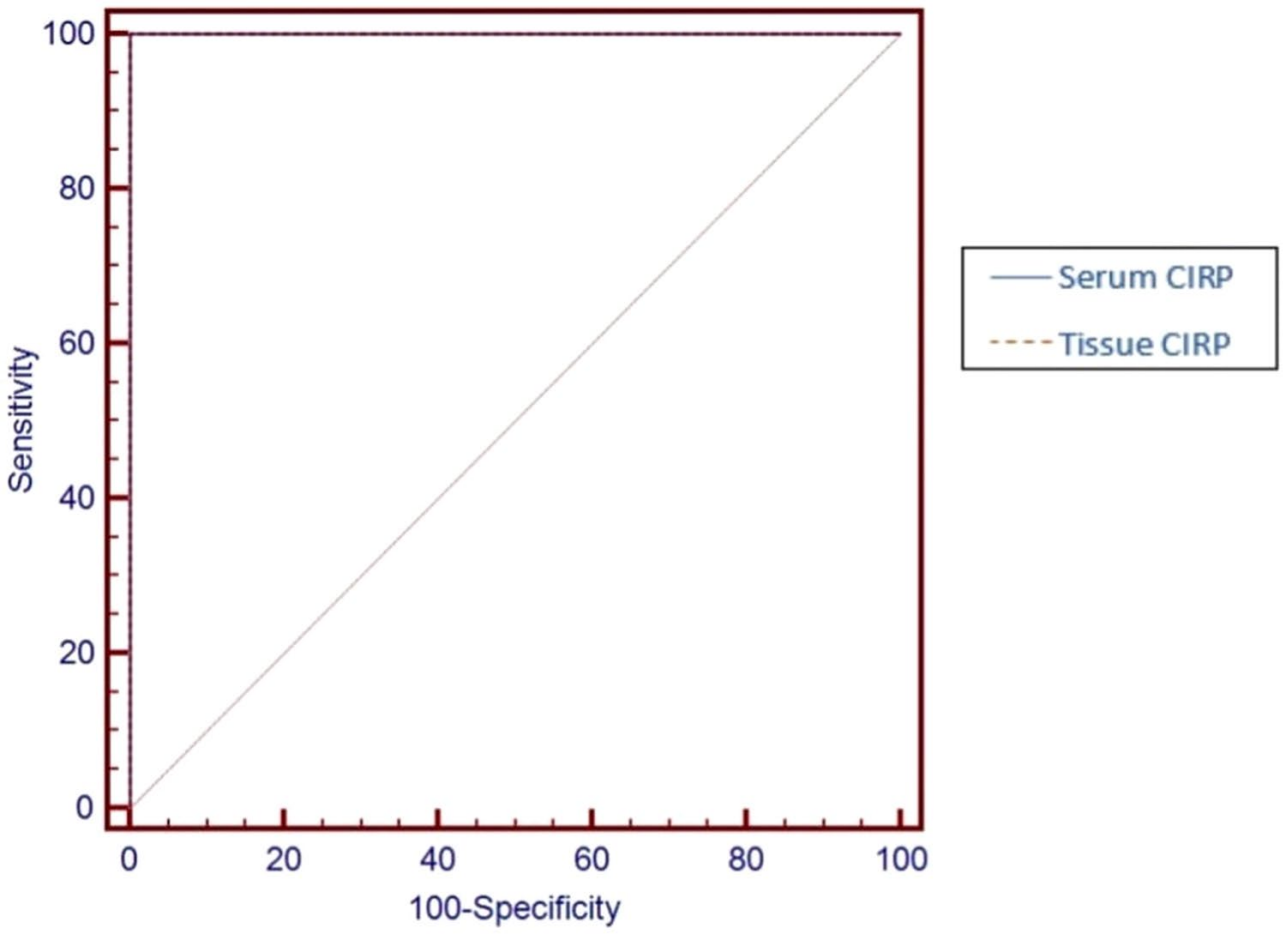
ROC curve of CIRP serum and tissue levels to differentiate between patients and healthy controls

The serum CIRP's ROC curve in NSV patients demonstrated a cut-off level of > 86.55 pg/ml; i.e. under this threshold, a person would likely be classified healthy, with a sensitivity of 100% and specificity of 100.0%. Also, the tissue CIRP's ROC curve in NSV patients demonstrated a cut-off level of > 124.3 5 pg/ml. The sensitivity was 100% and the specificity was 100.0%, as well (Fig. 1, Table 3).

**Table 3:** ROC data for CIRP serum and tissue levels to differentiate between patients and healthy controls

	Cut-off point	AUC	Sensitivity	Specificity	PPV	NPV
Serum CIRP (pg/ml)	> 86.5	1.000	100.00	100.00	100.0	100.0
Tissue CIRP (pg/ml)	> 124.3	1.000	100.00	100.00	100.0	100.0

*AUC* Area under the curve, *PPV* positive predictive value, *NPV* negative predictive value

**Fig. 2:**
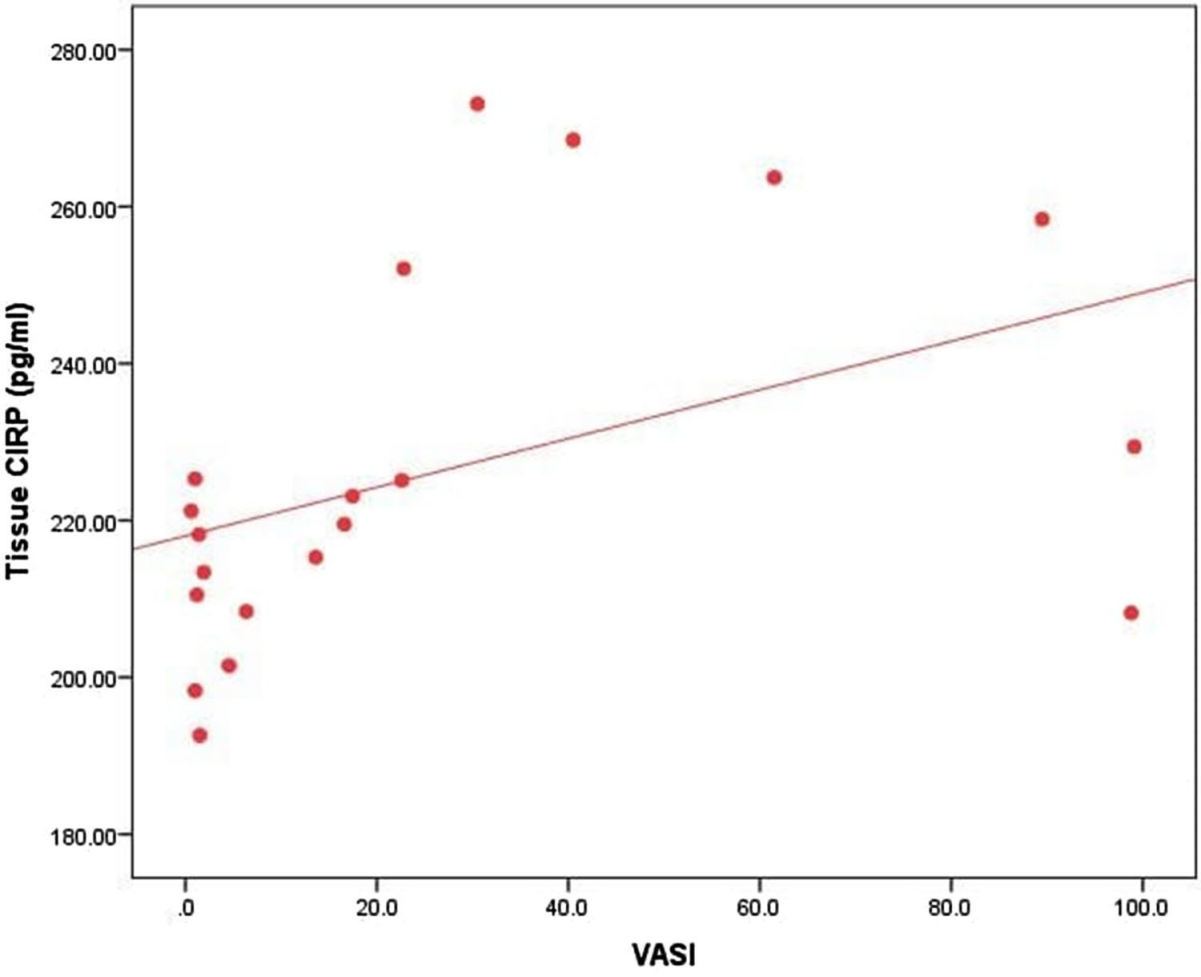
Correlation between tissue CIRP level and VASI

**Fig. 3:**
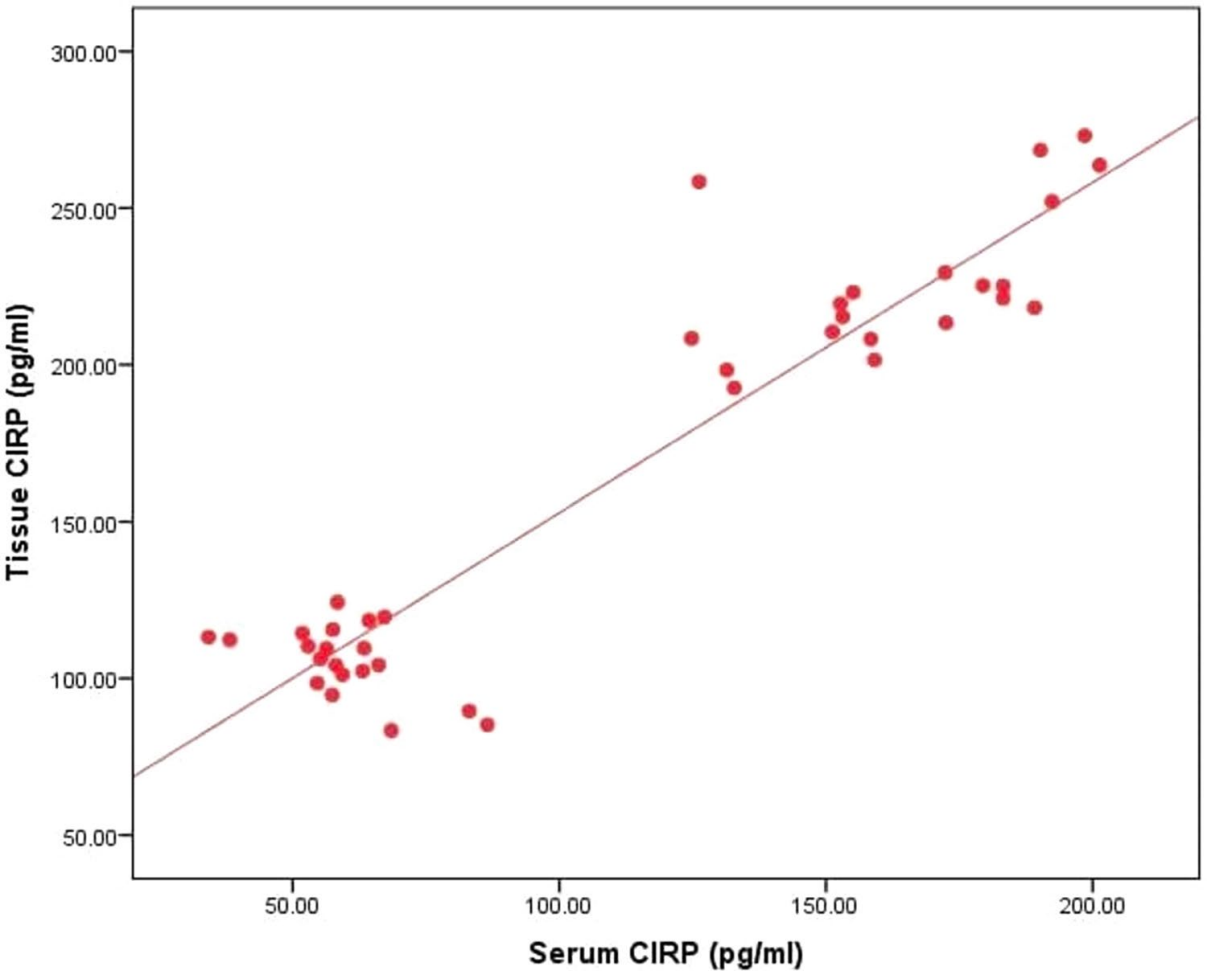
Correlation between tissue CIRP level and serum CIRP level

Serum CIRP did not show any statistically significant correlations with patient clinical parameters. A statistically significant positive correlation was found between tissue CIRP level and VASI score (*r* = 0.539, *P* = 0.014). No statistically significant correlation was found between tissue CIRP and clinical characteristics in patients, although. Moreover, a statistically significant positive correlation was discovered between serum and tissue CIRP levels (*r* = 0.641, *P* = 0.002) (Figs. 2, 3, Table 4).

**Table 4:** The Correlation between (CIRP serum and tissue levels) with different studied parameters in NSV patients

Parameters	Serum CIRP (pg/ml)	Tissue CIRP (pg/ml)
Age		
*r*	0.218	0.246
*P*	0.355	0.295
Duration (years) of Vitiligo		
*r*	− 0.078	0.278
*P*	0.744	0.235
VASI score		
*r*	0.215	0.539
*P*	0.362	0.014*
VIDA score		
*r*	− 0.215	− 0.058
*P*	0.363	0.810
Serum CIRP vs. tissue CIRP		
*r*	0.641	
*P*	0.002*	

*CIRP* cold-inducible RNA-binding protein, *VASI* vitiligo area scoring index, *VIDA* vitiligo disease activity score, *r* correlation coefficient

*P ≤ 0.05 is considered statistically significant

## Discussion

Although the vitiligo precise etiology is still debated, there is an agreement on the autoimmune theory of vitiligo [3]. Perilesional inflammatory infiltrate in vitiligo consists of both CD4 + & CD8 + cells, often with an increased CD8 + /CD4 + ratio. These cells exhibit predominantly T helper-1 cytokine profile, with the marked release of IFN-γ and TNF-α [13].

Being a DAMP molecule, CIRP is thought to promote inflammation resulting in tissue damage. Previous research showed that in sepsis, CIRP adheres to the TLR4/MD2 complex on CD8 + and CD4 + T-cells, resulting in their activation [14] and the production of TNFα and HMGB1 from APCs [8].

To learn about its potential role as a key factor in vitiligo, we investigated the serum and tissue levels of CIRP in vitiligo patients and healthy individuals. In this study, patients had considerably elevated levels of CIRP in serum and tissue than the healthy controls. In addition, the results of the ROC curve demonstrated that CIRP serum and tissue levels can be used to distinguish between patients and healthy controls with high sensitivity and specificity.

In addition, we found that serum CIRP didn’t show any significant correlation with the different clinical parameters, while tissue CIRP level was elevated in patients with higher VASI score significantly. Thus, the present results denote that CIRP could be a sign of vitiligo extension since tissue CIRP is significantly correlated with VASI score.

This is the first study to our knowledge to evaluate the role of CIRP in NSV. It has been shown that CIRP level is raised in various autoimmune and inflammatory diseases. Yoo et al. [15] reported that CIRP in rheumatoid arthritis(RA) was higher in both the synovia and serum and correlated with the disease's activity, which identified CIRP as a possible indicator of RA disease activity.

Shin et al. [16] found that alopecia areata (AA) patients had considerably greater serum levels of CIRP than the healthy group. In addition, showed a correlation between the serum CIRP and both disease duration and activity. Both RA and AA are autoimmune disorders and were associated with vitiligo in multiple genetic and epidemiological researches [4].

The initial event in melanocytes destruction could be oxidative stress [3]. In the current study, extracellular CIRP serum levels, which are released extracellularly under stress, were elevated in patients with NSV suggesting that extracellular CIRP acts as a candidate bridge between external stress and the skin's innate immune response.

In addition to inducing cultured macrophages to release TNF-α, HMGB1and IL-6, [7] It was demonstrated that CIRP stimulates inflammation via the NF-κB protein complex and stimulates TNF-α, IL-8 and IL-1β release in cultured fibroblasts [17]. IL-1β, HMGB1, IL-8, and IL-6 TNF-α are believed to have a crucial role in the innate immune response, and their levels were considerably increased in vitiligo patients' serum and skin [10, 18].

Research has shown that CIRP triggers the activation of NLRP3 inflammasome, which triggers IL-1β and caspase-1 release [19]. NLRP3 expression has been elevated in perilesional vitiligo skin, which correlates with increased cutaneous IL-1β expression and disease severity [20]. Thus, we hypothesize that increased CIRP might stimulate the activation of NLRP3 inflammasome that subsequently induce IL-1β involved in vitiligo pathogenesis.

Studies showed a positive correlation between serum and tissue CIRP concentrations in a number of inflammatory and ischemic conditions. The concentrations of CIRP in tissue and serum were higher in hepatic and renal ischemia–reperfusion mice models [21, 22]. Similar results were reported in sepsis [7], rheumatoid arthritis [15] and chronic inflammatory disorders such as (COPD) patients [23]. These studies agree with our finding of a positive association between serum and tissue CIRP levels in NSV patients.

Bazid et al. [24] showed that CIRP levels were likewise increased in psoriasis patients compared with controls. Despite the fact that CIRP levels in the serum and epidermis were positively correlated, its expression in epidermis was lower in psoriatic patients than healthy controls. Their results demonstrated that CIRP is critical for the pathogenicity of psoriasis, with variations between its concentrations inside and outside the cells indicating a possible difference in how it functions.

Extracellular CIRP could represent a hope for inflammatory diseases therapy. C23, a CIRP-derived small peptide, was found to decrease ICAM-1 expression and IL-1β secretion in lung endothelial cells activated by recombinant CIRP in rats [25] additionally, CIRP- neutralizing antibodies were found to inhibit TNF-α production in macrophages induced by recombinant CIRP [7]. Therefore, CIRP could be identified as a new therapeutic target for NSV.

The small sample size, besides the inability to determine the intracellular CIRP expression, represents the main limitations of the current study. Therefore, further large-scale studies with immunohistochemistry are needed.

## Conclusion

Increased serum and tissue CIRP levels in NSV patients, pointing to a possible CIRP role in NSV pathophysiology. CIRP could be studied as a promising marker for the extension of vitiligo since tissue CIRP is significantly correlated with VASI score. Further large-scale studies are required to confirm these preliminary results and examine the relationship between CIRP and disease activity.

What’s already known about this topic? •DAMPs have been involved in the pathogenesis of vitiligo.

•Extracellular CIRP may function as a DAMP.

What does this study add? •Serum and tissue CIRP levels elevated in patients with NSV suggesting its role in vitiligo pathogenesis .

## Data Availability

All data concerning this study is available upon request.
