# Correction to: Evaluation of serum and tissue levels of cold‑inducible RNA‑binding protein in non‑segmental Vitiligo

**DOI:** 10.1007/s00403-023-02653-y

**Published:** 2023-06-16

**Authors:** Nayera Hassan Moftah, Huda Alnos, Laila Rashed, Mervat Hamdino

**Affiliations:** 1 Dermatology and Venereology Department, Faculty of Medicine for Girls, Al-Azhar University, Cairo, Egypt; 2 Biochemistry Department, Faculty of Medicine, Cairo University, Cairo, Egypt

**Correction to: Archives of Dermatological Research** 10.1007/s00403-023-02586-6

In this article the wrong figure appeared as Fig. 2; the Fig. 2 should have appeared as shown below.

**Fig. 2:**
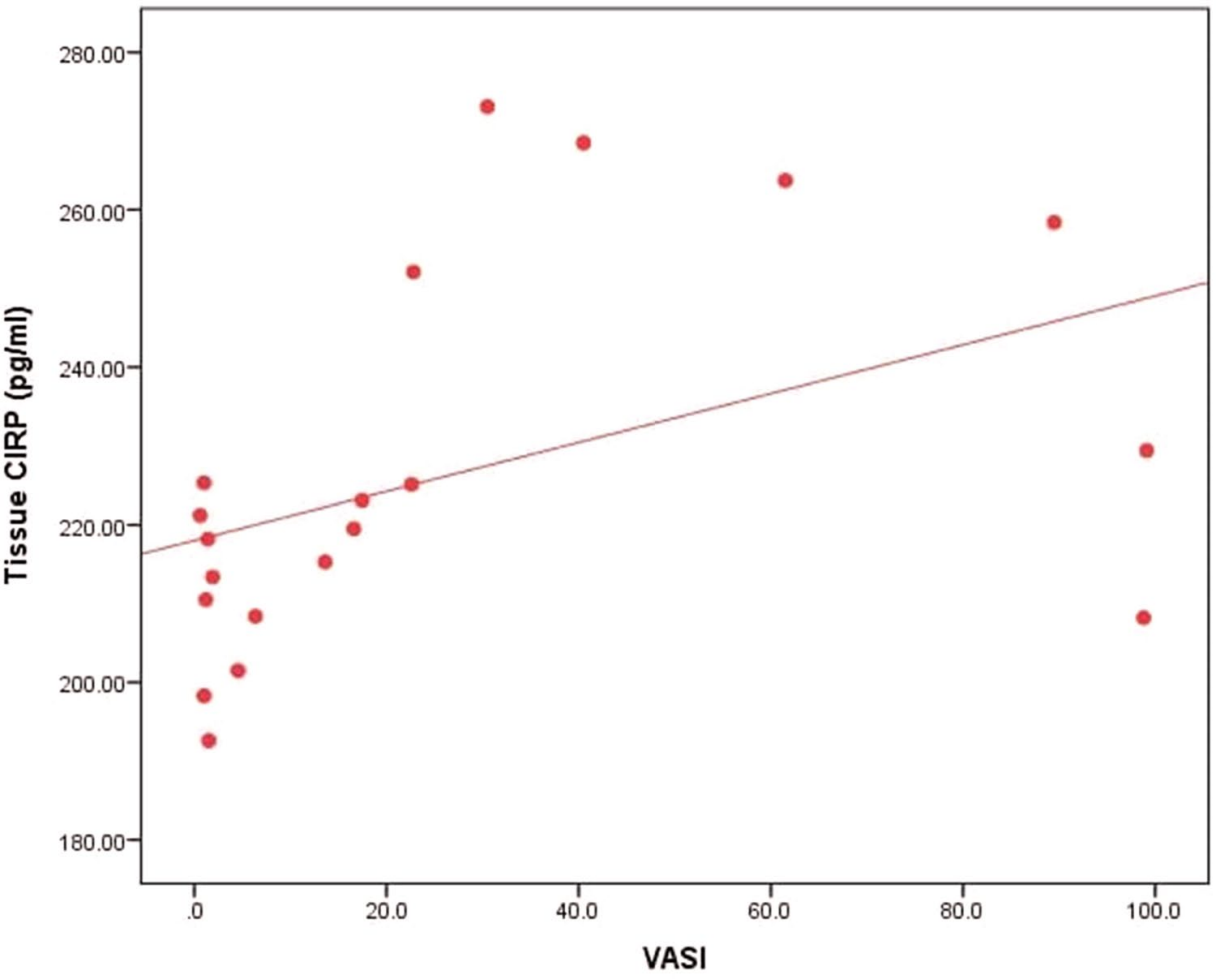
Correlation between tissue CIRP level and VASI

The original article has been corrected.

